# Antiseizure medications for post‐stroke epilepsy: A real‐world prospective cohort study

**DOI:** 10.1002/brb3.2330

**Published:** 2021-08-22

**Authors:** Tomotaka Tanaka, Kazuki Fukuma, Soichiro Abe, Soichiro Matsubara, Rie Motoyama, Masahiro Mizobuchi, Hajime Yoshimura, Takayuki Matsuki, Yasuhiro Manabe, Junichiro Suzuki, Shuhei Ikeda, Naruhiko Kamogawa, Hiroyuki Ishiyama, Katsuya Kobayashi, Akihiro Shimotake, Kunihiro Nishimura, Daisuke Onozuka, Masatoshi Koga, Kazunori Toyoda, Shigeo Murayama, Riki Matsumoto, Ryosuke Takahashi, Akio Ikeda, Masafumi Ihara, for the PROPOSE Study Investigators

**Affiliations:** ^1^ Department of Neurology National Cerebral and Cardiovascular Center Osaka Japan; ^2^ Department of Neurology, Graduate School of Medical Sciences Kumamoto University Kumamoto Japan; ^3^ Department of Neurology Tokyo Metropolitan Geriatric Hospital and Institute of Gerontology Tokyo Japan; ^4^ Department of Neurology Nakamura Memorial Hospital Sapporo Japan; ^5^ Clinic of Minami‐ichijyo Neurology Sappro Japan; ^6^ Department of Neurology Kobe City Medical Center General Hospital Kobe Japan; ^7^ Department of Neurology St. Mary's Hospital Fukuoka Japan; ^8^ Department of Neurology National Hospital Organization Okayama Medical Center Okayama Japan; ^9^ Department of Neurology Toyota Memorial Hospital Toyota Japan; ^10^ Department of Neurology Kyoto University Graduate School of Medicine Kyoto Japan; ^11^ Departments of Preventive Medicine and Epidemiology National Cerebral and Cardiovascular Center Osaka Japan; ^12^ Department of Cerebrovascular Medicine National Cerebral and Cardiovascular Center Osaka Japan; ^13^ Division of Neurology Kobe University Graduate School of Medicine Kobe Japan; ^14^ Department of Epilepsy, Movement Disorders and Physiology Kyoto University Graduate School of Medicine Kyoto Japan

**Keywords:** antiseizure medication, post‐stroke epilepsy, retention, seizure recurrence, tolerability

## Abstract

**Background and purpose:**

The management of post‐stroke epilepsy (PSE) should ideally include prevention of both seizure and adverse effects; however, an optimal antiseizure medications (ASM) regimen has yet been established. The purpose of this study is to assess seizure recurrence, retention, and tolerability of older‐generation and newer‐generation ASM for PSE.

**Methods:**

This prospective multicenter cohort study (PROgnosis of Post‐Stroke Epilepsy [PROPOSE] study) was conducted from November 2014 to September 2019 at eight hospitals. A total of 372 patients admitted and treated with ASM at discharge were recruited. Due to the non‐interventional nature of the study, ASM regimen was not adjusted and followed standard hospital practices. The primary outcome was seizure recurrence in patients receiving older‐generation and newer‐generation ASM. The secondary outcomes were the retention and tolerability of ASM regimens.

**Results:**

Of the 372 PSE patients with ASM at discharge (median [IQR] age, 73 [64–81] years; 139 women [37.4%]), 36 were treated with older‐generation, 286 with newer‐generation, and 50 with mixed‐generation ASM. In older‐ and newer‐generation ASM groups (*n* = 322), 98 patients (30.4%) had recurrent seizures and 91 patients (28.3%) switched ASM regimen during the follow‐up (371 [347–420] days). Seizure recurrence was lower in newer‐generation, compared with the older‐generation, ASM (hazard ratio [HR], 0.42, 95%CI 0.27–0.70; *p* = .0013). ASM regimen withdrawal and change of dosages were lower in newer‐generation ASM (HR, 0.34, 95% CI 0.21–0.56, *p* < .0001).

**Conclusions:**

Newer‐generation ASM possess advantages over older‐generation ASM for secondary prophylaxis of post‐stroke seizures in clinical practice.

## Graphical abstract

The current study is a real‐world prospective cohort study to evaluate seizure control, retention, and tolerability of older or newer‐generation anti‐seizure medication (ASM) treatment in post‐stroke epilepsy (PSE). Seizure recurrence was lower in newer‐generation, compared with the older‐generation, ASM (hazard ratio [HR], 0.42, 95%CI 0.27–0.70; *p* = .0013). ASM regimen withdrawal and change of dosages were lower in newer‐generation ASM (HR, 0.34, 95% CI 0.21–0.56, *p* < .0001). These findings suggest potential for newer‐generation ASM as the primary choice in the secondary prophylaxis of PSE.

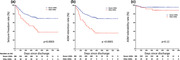

## 1 INTRODUCTION

Post‐stroke epilepsy (PSE), one of the major sequelae of stroke, is the most common cause of epilepsy in the elderly (Sen et al., 2020). Stroke survivors with potential risk of PSE have been increasing (Tanaka & Ihara, 2017) as recent advances in acute stroke treatments have dramatically improved mortality rate. Antiseizure medications (ASMs) are the mainstay in seizure control in PSE, with most patients generally well controlled by a single dose of ASM (Ryvlin et al., 2006); however, approximately one‐third of PSE patients experience seizure recurrence under ASM treatment within 1 year (Tanaka et al., 2015), and another study also demonstrated that approximately 20% of patients with PSE developed pharmaco‐resistance and exhibited associations with younger age at stroke onset, stroke type and severity, status epilepticus occurrence, and seizure type (Lattanzi et al., 2021). These results may lead to heightened anxiety regarding seizure recurrence and suppressed social activities in stroke survivors.

According to American and European guidelines (Holtkamp et al., 2017; Winstein et al., 2016 ), use of ASM for secondary prophylaxis of post‐stroke seizures has been recommended and newer‐generation ASM, levetiracetam (LEV) and lamotrigine (LTG), have been suggested as first‐line treatments due to fewer adverse effects (AE) (Ferlazzo et al., 2016; Feyissa et al., 2019).

Despite such recommendations, there is currently no established evidence on whether newer‐generation ASM are suitable for prevention of PSE (Tanaka & Ihara, 2017). To date, two small randomized control studies (RCT) have specifically evaluated ASM in PSE (Brigo et al., 2018). In a nationwide, population‐based study, Huang et al. reported valproic acid (VPA) and newer‐generation ASM have superior seizure control compared to phenytoin (PHT) (Huang et al., 2015); however, this study was retrospective and was based on information from a health insurance database. Furthermore, a randomized control study conducted by Werhann et al. found elderly patients with new‐onset focal epilepsy (originated from stroke in 65.9%), showed superior tolerability and higher drug retention of LEV and LTG compared to controlled‐released carbamazepine (CBZ) (Werhahn et al., 2015); however, no significant differences were found in seizure freedom rates.

The objectives of the current study were to assess whether newer‐generation ASM have advantages over older‐generation ASM in terms of seizure control, retention, and tolerability in PSE over a 1‐year period in a real‐world clinical practice.

## 2 METHODS

### 2.1 Study design and patients

The PROgnosis of Post Stroke Epilepsy (PROPOSE) study was a multicenter, noninterventional, prospective observational cohort study. Patients hospitalized with PSE between November 2014 and September 2018 were recruited from eight hospitals in Japan. According to the new clinical definition from the International League Against Epilepsy (ILAE) (Fisher et al., 2014), one unprovoked seizure more than 7 days (late seizure) after index stroke was diagnosed as PSE. The median of the period between stroke onset and late seizure is 430 days. We excluded patients with only acute symptomatic seizure within 7 days of stroke onset, history of only asymptomatic stroke, another cause of epilepsy, or potentially epileptogenic comorbidities (intracranial tumors, traumatic brain injury, alcohol or drug abuse, or other probable causes). All diagnoses of PSE were confirmed by board‐certified epileptologists or neurologists at each hospital based on electroencephalogram (EEG, conducted in 375 [95.7%]), single photon emission computed tomography (SPECT, 152 [38.8%]), magnetic resonance imaging (MRI, 308 [78.6%]), as well as detailed seizure semiology during admission. The study was approved by our institutional ethical committee (M26‐093‐7) and each participating institutional review board and conducted in accordance with relevant institutional guidelines. Waiver of informed consent was granted by the ethical committees according to the “opt‐out” principle.

### 2.2 Clinical characteristics

Demographics, including age, sex, body weight, cardiovascular risk factors (hypertension, dyslipidemia, diabetes, smoking, chronic kidney diseases, atrial fibrillation), history of dementia, craniotomy, alcohol drinking, family history of epilepsy, and ASM medication history, were collected during admission. Index stroke were categorized into four types, ischemic stroke, intracranial hemorrhage, subarachnoid hemorrhage, and transient ischemic attack, and assessed for cortical stroke lesions (frontal, parietal, temporal, occipital lobe) and size in major axis (categorized as less than 15 mm, 15−30 mm, more than 30 mm) by computed tomography (CT) or MRI. Functional disability was evaluated at discharge based on modified Rankin Scale. We also studied the histories of early (within 7 days post stroke) and late (more than 7 days) seizures.

PSE seizures were classified as focal aware seizure, focal impaired awareness seizure, focal‐to‐bilateral tonic‐clonic seizure, and others, according to the ILAE classification of seizures (Fisher et al., 2017). Semiological information of the ictal and post‐ictal phase, such as motor seizure, ictal paresis, aphasia, amnesia, and the other semiologies, were collected. Standard scalp EEG was performed for more than 20 min after admission and analyzed for focal and rhythmic slow wave, periodic discharge, and paroxysmal activity. EEG terminologies were based on the Salzburg EEG criteria (Hirsch et al., 2013). Ictal or postictal SPECT and diffusion‐weighted MRI (DWI) with apparent diffusion coefficient (ADC) were performed for identifying a hyperperfusion area in endorsing the PSE diagnosis, based on our previous report (Fukuma et al., 2020).

### 2.3 ASM seizure control, retention, and tolerability assessments

**FIGURE 1:**
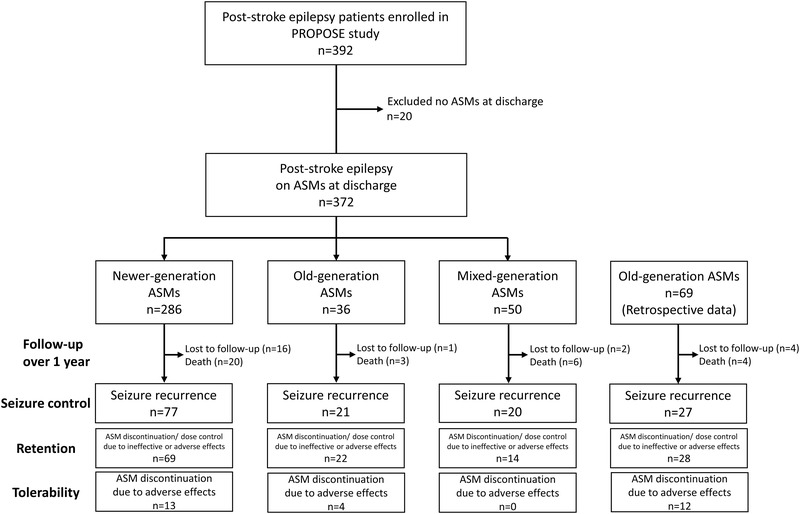
Participant flowchart of study cohorts. After exclusion of cases without antiseizure medication (ASM), 372 post‐stroke epilepsy patients were prospectively followed up for incident seizure recurrence, withdrawal, and change of dosages due to adverse effects or inadequate seizure control (retention) and withdrawal due to adverse effects (tolerability) (See Figure 2). For verification analysis, the retrospective older‐generation ASM cohort (*n* = 69) was obtained from the retrospective cohort database (See eFigure 2)

We evaluated the seizure control, retention, and tolerability of ASM treatment in the secondary prevention of seizure after discharge. We excluded patients without ASM prescription at discharge. For secondary prophylaxis of seizures, PSE patients were categorized into three groups (Figure 1): older‐generation (*n* = 36), newer‐generation (*n* = 286), and mixed generation, ASM groups (*n* = 50). CBZ, VPA, PHT, phenobarbital (PB), clonazepam (CZP), and clobazam (CLB) were defined as “older‐generation ASM,” and LEV, LTG, lacosamide (LCM), zonisamide (ZNS), perampanel (PER), gabapentin (GBP), and topiramate (TPM) as “newer‐generation ASM,” which were launched in Japan after 2006. A “mixed‐generation” ASM group, defined as a combination of older‐ and newer‐generation ASM, was excluded from the main analysis due to various factors on the choice of ASM regimen. These definitions were determined regardless of the number of ASM and selection of regimen was not altered due to the non‐interventional nature of the current study, which followed standard hospital practices. The dosage of each ASM, as well as information on whether the serum concentration of older‐generation ASM reached an adequate therapeutic range at discharge, were collected. After a baseline dataset was collected during admission period, each participant was followed‐up according to usual clinical practice in each hospital over a 1‐year period. The mixed‐generation ASM group was excluded from the main analyses.

The primary outcome was seizure freedom rate over the complete follow‐up period since discharge. The secondary outcomes were the time to discontinuation or dosage adjustment of initial ASM regimen due to ineffectiveness or intolerable ASM‐related AE (defined as ASM retention) and time to discontinuation of initial ASM regimen due to intolerable ASM‐related AE (defined as ASM tolerability, a subgroup of ASM retention outcome). The incidence of discontinuation or dosage adjustment due to poor adherence or other reasons were not considered outcomes. Board‐certified epileptologists or neurologists closely assessed episodes of first unprovoked seizure recurrence, ASM regimens, and reasons behind ASM discontinuation or dosage adjustment from hospitalization and/or outpatient visit records and seizure diaries with face‐to‐face consultations. If outpatient visits were not feasible, assessment was made through telephone interview with patients, relatives, or general physicians at 6 and 12 months. Patients who died or were lost to follow‐up were assessed as of the last visit. The maximum period of observation until the last follow‐up was 800 days.

### 2.4 Verification

In the current prospective study, the older‐generation ASM group comprised just 36 patients, presumably owing to the trend of using newer‐generation ASM. LEV was predominantly prescribed in preference to older‐generation ASM in Japan (Nakamura et al., 2020), as typically also found in other countries (Glauser et al., 2013). To compensate for the imbalance in the number between groups and to verify the results, we added older‐generation ASM cases (*n* = 69) from the retrospective PSE cohort database of our institution between January 2011 to October 2014, comprising 15 newer‐generation, 18 mixed‐generation, and 69 older‐generation ASM. Retrospective data were obtained from registered PSE patients treated and followed up after discharge completely at our hospitals. During this period, most newer‐generation ASM monotherapies were not covered by Japanese public health insurance.

### 2.5 Statistical analyses

Data are presented as median (interquartile range [IQR]) or number (%). Variables were compared using Wilcoxon's test for continuous variables and Pearson's chi‐square test for categorical variables. Time to first seizure recurrence (seizure freedom rates), discontinuation or dosage adjustment (ASM retention rates) and discontinuation (ASM tolerability rates) between older‐ and newer‐generation ASM groups was analyzed by the Kaplan–Meier method and compared by log‐rank test. Follow‐up time was defined as the period from discharge until death, loss to follow‐up or final follow‐up visit. Patients who withdrew due to the above reasons were censored at the time of withdrawal, and those who did not withdraw were censored at their last visit. We also determined cumulative seizure freedom rates by 90 days, 180 days, and 1 year after discharge.

Cox proportional hazards modeling was used to calculate hazard ratios (HR) with 95% confidence intervals (CI). Potential confounding factors such as sex (Kim et al., 2016), age (Kim et al., 2016; Tanaka et al., 2015; Tomari et al., 2017), and other variables with a *p* value < .10 between the ASM groups were adjusted in a multivariate model (model 1: age and sex; model 2: age, sex, dyslipidemia, dementia, temporal lobe stroke lesion, and spike or sharp wave on EEG). In addition, we conducted additional analyses by eliminating data on seizure outcomes of PSE patients with inadequate or unknown concentrations of older‐generation ASM (*n* = 18).

As with verification analyses, prospective newer‐generation ASM were compared against the retrospective older‐generation ASM group, or the combination of prospective and retrospective older‐generation ASM groups, using the same methods. Two‐sided values of *p* < .05 were considered significant. All statistical analyses were performed with the JMP 12.2.0 software package (SAS Institute Inc., Cary, NC, USA). All data were anonymized, stored, and fixed in an electronic data capture system by the data manager prior to analysis.

## 3 RESULTS

From November 2014 to September 2018, a total of 392 patients with a diagnosis of PSE in the eight sampled hospitals met the eligibility criteria and were prospectively enrolled into the PROPOSE study (Figure 1). Of these, we excluded 20 patients not receiving ASM at discharge. Of the remaining 372 patients, 36 were treated with older‐generation, 286 with newer‐generation, and 50 with mixed‐generation, ASM as a secondary prophylaxis.

The demographics and clinical course characteristics of older‐ and newer‐generation ASM groups are shown in Table 1. The basis for diagnosis of PSE, including seizure semiology and epileptic examination findings, are described in Table 2. Of these 322 patients (excluding mixed‐generation ASM group), 111 (34.5%) had a previously diagnosed PSE and 90 (28.0%) had received ASM therapy before admission. PSE etiologies of stroke subtype were ischemic stroke (including 93 [28.9%] cardiogenic embolism) in 199 (61.8%), intracerebral hemorrhage in 114 (35.4%), subarachnoid hemorrhage in 21 (6.5%), transient ischemic attack in 3 (0.9%), and overlapped stroke types in 15 (4.7%). On admission for PSE, 185 (57.5%) patients exhibited focal‐to‐bilateral tonic‐clonic seizure and 236 (73.3%) with focal or generalized motor seizure. Clinical findings revealed 104 (33.5%) of 310 had a spike or sharp wave on EEG, 75 (61.0%) of 123 had hyperperfusion lesions on SPECT, and 73 (29.2%) of 250 had seizure‐related hyperintensities on DWI. Baseline demographics, clinical manifestations, and findings were generally comparable between the two treatment groups (Table 1). There was no difference in percentages of one generation ASM polytherapy at discharge (older‐generation ASM, 4 [11.1%]; newer‐generation ASM, 15 [5.2%]). Between older and newer‐generation ASM, the most common ASM was LEV (*n* = 263), followed by CBZ (*n* = 26), LCM (*n* = 18), and VPA (*n* = 9). The regimen and mean daily dosages of ASM, and proportion of patients with adequate serum concentrations of older‐generation ASM at discharge are described in eTables 1 and 2.

**TABLE 1:** Patient characteristics

	All (*n* = 322)	Older‐generation ASM (*n* = 36)	Newer‐generation ASM (*n* = 286)	*p* Value
Age, years, median (IQR)	74 (65–82)	75.5 (70–83.8)	74 (64.8–81.3)	.20
Female (%)	127 (39.4)	14 (39)	113 (39.5)	.94
Body weight, kg, median (IQR)	54 (45–63)	56 (47.3–67.3)	54 (44.5–63)	.49
Current alcohol consumption (%)	42 (13.0)	4 (11)	38 (13.3)	.71
Family history of epilepsy (%)	6 (1.9)	1 (3)	5 (1.8)	.67
Previous PSE (%)	111 (34.5)	13 (36)	98 (34.3)	.83
Previous ASM (%)	90 (28.0)	9 (25)	81 (28.3)	.68
Comorbidities				
Hypertension (%)	257 (79.8)	32 (89)	225 (78.7)	.15
Dyslipidemia (%)	151 (46.9)	22 (61)	129 (45.1)	.070
Diabetes (%)	80 (24.8)	10 (28)	70 (24.5)	.67
Chronic kidney diseases (%)	121 (37.6)	12 (33)	109 (38.1)	.58
Liver cirrhosis (%)	6 (1.9)	0 (0)	6 (2.1)	.38
Atrial fibrillation (%)	112 (34.8)	14 (39)	98 (34.3)	.58
Dementia (%)	111 (34.5)	17 (47)	94 (32.9)	.088
Craniotomy (%)	48 (14.9)	5 (14)	43 (15.0)	.86
Etiology of stroke subtype (%)				
Infarction (%)	199 (61.8)	25 (69)	174 (60.8)	.32
Cardiac embolism (%)	93 (28.9)	13 (36)	80 (28.0)	.31
Hemorrhage (%)	114 (35.4)	10 (28)	104 (36.4)	.31
TIA (%)	3 (0.9)	0 (0)	3 (1.1)	.54
SAH (%)	21 (6.5)	2 (6)	19 (6.6)	.80
Previous early seizure^a^ (%)	18 (6.8)	2 (6)	16 (6.9)	.89
Stroke lesion				
Frontal lobe (%)	82 (25.5)	8 (22)	74 (25.9)	.64
Parietal lobe (%)	168 (52.2)	20 (56)	148 (51.8)	.67
Temporal lobe (%)	137 (42.5)	20 (56)	117 (40.9)	.094
Occipital lobe (%)	53 (16.5)	7 (19)	46 (16.1)	.61
Cortical lesion (%)	262 (81.4)	31 (86)	231 (80.8)	.44
Stroke size				.97
<15 mm (%)	38 (11.8)	4 (11)	34 (11.9)	
15−30 mm (%)	66 (20.5)	7 (19)	59 (20.6)	
>30 mm (%)	218 (67.7)	25 (70)	193 (67.5)	
Clinical course during hospitalization				
Classification of seizures				.32
Focal aware seizure (%)	38 (11.8)	1 (3)	37 (12.9)	
Focal impaired aware seizure (%)	97 (30.1)	12 (33)	85 (29.7)	
Focal‐to‐bilateral tonic‐clonic seizure (%)	185 (57.5)	23 (64)	162 (56.6)	
Others (%)	2 (0.6)	0 (0)	2 (0.7)	
Detail of seizure				
Focal or generalized motor seizure (%)	236 (73.3)	29 (81)	207 (72.4)	.30
Weakness (%)	66 (20.5)	6 (17)	60 (21)	.55
Aphasia (%)	59 (18.3)	5 (14)	54 (18.9)	.47
Amnesia (%)	7 (2.2)	0 (0)	7 (2.5)	.34
EEG findings *n* = 310^b^				
Focal slow wave (%)	272 (87.7)	29 (88)	243 (87.7)	.98
Spike/Sharp wave (%)	104 (33.5)	16 (49)	88 (31.8)	.06
Rhythmic slow wave (%)	38 (12.3)	2 (6)	36 (13.0)	.25
Periodic discharge (%)	52 (16.8)	6 (18)	46 (16.6)	.82
Hyperperfusion on SPECT (%) *n* = 123^a^	75 (61.0)	6 (55)	69 (61.6)	.65
Seizure‐related hyperintensity on DWI (%) *n* = 250^a^	73 (29.2)	10 (32)	63 (28.8)	.69
Hospitalization period, days, median (IQR)	9 (6–16)	8.5 (6.3–16.3)	9 (5.8–16)	.90
mRS at discharge, median (IQR)	3 (2–4)	3 (2–4)	3 (2–4)	.96
ASM combination therapy (%)	19 (5.9)	4 (11)	15 (5.2)	.16

Abbreviations: ASM, antiseizure medication; DWI, diffusion weighted image; EEG, electroencephalogram; IQR, interquartile range; mRS, modified Rankin Scale.; NIHSS, National Institutes of Health Stroke Scale; PSE, post‐stroke epilepsy; SAH, subarachnoid hemorrhage; SPECT, single photon emission computed tomography; TIA, transient ischemic attack.

Data are presented as median (interquartile range) or absolute (percentage) values.

Mixed generation ASM group (*n* = 50) was excluded in the table.

^a^ Lack of information on history of early seizure in 58 cases because of earlier admission in a different hospital.

^b^ EEG was conducted in 310, SPECT in 123, MRI in 250 in the acute stage of index seizure during hospitalization.

**TABLE 2:** Basis of diagnosis for PSE in prospective cohort (*n* = 322)

Focal or generalized motor seizure^a^ *n* = 236			
**Features of seizure**		**Supporting epileptic findings**	
Disturbance of consciousness	183 (77.5%)	EEG +^b^, MRI +^c^, SPECT +^d^	7 (3.0%)
Fluctuating consciousness	61 (25.8%)	EEG +, MRI +	24 (10.2%)
Tonic‐clonic seizure	168 (71.2%)	EEG +, SPECT +	14 (5.9%)
Tonic seizure	57 (24.2%)	EEG +	49 (20.8%)
Myoclonic seizure	11 (4.7%)	MRI +, SPECT +	6 (2.5%)
Eye deviation	125 (53.0%)	SPECT +	21 (8.9%)
Ictal emotional expression	6 (2.5%)	MRI +	22 (9.3%)
Autonomic symptom	6 (2.5%)	No remarkable findings	93 (39.4%)
Ictal paresis	43 (18.2%)		
Ictal aphasia	28 (11.9%)		
Ictal amnesia	2 (0.8%)		
**Non‐motor seizure *n* = 86**			
**Features of seizure**		**Supporting epileptic findings**	
Disturbance of consciousness	70 (81%)	EEG +, MRI +, SPECT +	3 (3%)
Fluctuating consciousness	35 (41%)	EEG +, MRI +	4 (5%)
Eye deviation	28 (33%)	EEG +, SPECT +	13 (15%)
Ictal emotional expression	2 (2%)	EEG +	17 (20%)
Hallucination	3 (3%)	MRI +, SPECT +	2 (2%)
Autonomic symptom	1 (1%)	SPECT +	9 (10%)
Abnormal sensations	1 (1%)	MRI +	5 (6%)
Ictal paresis	21 (24%)	No remarkable findings^a^	33 (38%)
Visual impairment	5 (6%)		
Deafness	1 (1%)		
Ictal aphasia	28 (33%)		
Ictal amnesia	4 (5%)		

EEG was performed once in 310 (96.3%) cases and twice in 90 cases, MRI (DWI) in 250 (77.6%), and SPECT in 123 (38.2%).

^a^ Focal or generalized motor seizure was defined as tonic‐clonic, tonic, or myoclonic seizure.

^b^ EEG + means the presence of spike or sharp wave, periodic discharge, or rhythmic slow wave on scalp EEG. EEG terminologies were based on the American Clinical Neurophysiology Society's Standardized Critical Care EEG Terminology: 2012 version (Hirsch et al., 2013).

^c^ MRI + means the presence of hyperintensities that are cortically based with an atypical vascular distribution and mostly reversible on DWI (not stroke) (Koksel et al., 2018).

^d^ SPECT + means the presence of ictal or postictal hyperperfusion area on SPECT. The details of identification of hyperperfusion are described in our previous report (Fukuma et al., 2020).

### 3.1 Primary outcome: Seizure recurrence

**FIGURE 2:**
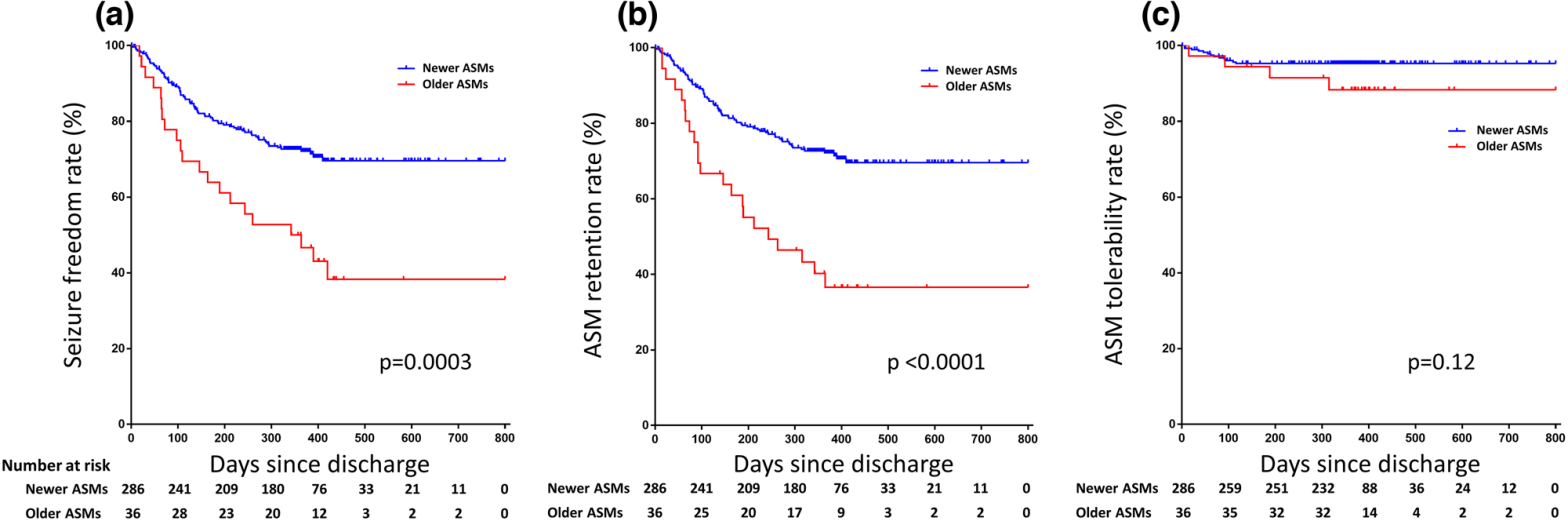
Kaplan–Meier curves of prospective newer‐generation ASM (*n* = 286) versus prospective older‐generation ASM (*n* = 36) for seizure recurrence, retention, and tolerability of ASM. (a) Time to seizure recurrence between older‐ and newer‐generation ASM. (b) Time to ASM discontinuation and dosage adjustment of initial ASM regimen due to ineffective or ASM‐related adverse effect to assess retention of ASM. (c) Time to discontinuation of initial ASM regimen due to ASM‐related adverse effects to assess tolerability of ASM. Censored values are indicated by dots. Mixed generation ASM group (*n* = 50) was excluded in the analyses

The median follow‐up period after index PSE was 371 (IQR, 347–420) days after discharge. The seizure freedom rates were 88.2% at 90 days, 78.3% at 180 days, 69.0% at 1 year, and 65.3% at the end of follow‐up (800 days). Within 1 year of discharge, 23 deaths and 17 difficulties of contact were recorded. The risk of recurrent seizure was significantly lower in newer‐generation, compared with the older‐generation, group (*p* = .0003, log‐rank) (Figure 2a).

The overall rates of seizure freedom were 38.3% with older‐generation, and 69.6% with newer‐generation, ASM. In Cox proportional hazards models adjusted for covariates, newer‐generation ASM had lower risk of seizure recurrence than older‐generation ASM; the crude HR was 0.42 (95% CI, 0.27−0.70), while adjusted HRs were 0.40 (95%CI, 0.25−0.67) in model 1 and 0.47 (95%CI, 0.29−0.81) in model 2 (Table 3). Even after PSE patients with inadequate or unknown concentration of older‐generation ASM (*n* = 18) were excluded from analysis, newer‐generation ASM retained a lower risk of seizure recurrence than older‐generation ASM (*p* < .0001) (eFigure 1).

**TABLE 3:** Cox regression analysis of seizure recurrence in newer‐generation ASM

**Prospective cohort *n* = 322**						
	**Unadjusted**		**Model 1** **^$^**		**Model 2** ^b^	
	HR (95%CI)	*p* Value	HR(95%CI)	*p* Value	HR(95%CI)	*p* Value
Seizure recurrence (ASM effectiveness)	0.42 (0.27−0.70)	.0013	0.40 (0.25−0.67)	.0008	0.47 (0.29−0.81)	.0075
Withdrawal of initial ASM regimens (ASM retention)^c^	0.34 (0.21−0.56)	<.0001	0.32 (0.20−0.53)	<.0001	0.35 (0.21−0.61)	.0003
Discontinuation of initial ASM (ASM tolerability)^d^	0.42 (0.15−1.50)	.16	0.42 (0.15−1.49)	.16	0.43 (0.13−1.90)	.23

Abbreviations: ASM, antiepileptic drug.; CI, confidence interval; HR, hazard ratio.

^a^ Model 1: age, sex.

^b^ Model 2: age, sex, dyslipidemia, dementia, temporal lobe stroke lesion, and spike or sharp wave on electroencephalogram.

c Withdrawal of initial ASM regimens (discontinue or change the dosage of ASM) due to ineffectiveness or ASM‐related adverse effect.

^d^ Discontinuation of initial ASM due to intolerable ASM‐related adverse effect.

### 3.2 Secondary outcomes: Retention and tolerability of ASM

During the same period, 36.5% patients in the older‐generation, and 72.0% patients in the newer‐generation ASM group maintained the initial ASM regimens. Withdrawal from the study was due to drug ineffectiveness and AE: 68.2% and 31.8% in the older‐generation, and 69.6% and 30.4% in the newer‐generation ASM. Compared with older‐generation, retention rate was significantly higher in the newer‐generation ASM (*p* < .0001, log‐rank) (Figure 2b). In the Cox proportional hazards models, adjusted HRs for newer‐generation ASM were 0.32 compared with older‐generation (95%CI, 0.20−0.53) in model 1 and 0.35 (95%CI, 0.21−0.61) in model 2 (Table 3).

The ASM tolerability rate tended to be higher in the newer‐generation ASM (Figure 2c), though there were no significant differences between the groups (*p* = .12). The most common AE leading to discontinuation or dosage adjustment were skin eruptions (*n* = 11), dizziness (*n* = 5), and behavioral changes (*n* = 5) (eTable 3). No patients reported severe AE leading to death.

### 3.3 Verification analysis: Prospective and retrospective data

To validate the results of this prospective study, verification analysis was conducted using a retrospective older‐generation ASM cohort (*n* = 69) (Figure 1). Overall, in the retrospective older‐generation cohort, 27 (39.1%) of 69 patients had recurrent seizure, 28 (40.6%) had discontinuation or dosage adjustment due to ineffectiveness or intolerable AE, and 12 (17.4%) withdrew the first ASM regimen due to intolerable AE during a follow‐up period of median 361 (IQR, 305–422) days. Patient characteristics of the retrospective older‐generation cohort are described in eTable 4. Compared with the prospective newer‐generation (*n* = 286), the retrospective older‐generation ASM cohort showed significantly lower seizure freedom (*p* = .036; eFigure 2A) and ASM retention (*p* = .0015; eFigure 2B). In Cox proportional modelling, newer‐generation ASM had similar trends toward lower risk of seizure recurrence (HR, 0.63, 95%CI, 0.41−0.99, *p* = .046) and high retention (HR, 0.50, 95%CI, 0.33−0.79, *p* = .0033). Of note, the retrospective cases revealed newer‐generation ASM had significantly lower risk in terms of tolerability than older‐generation ASM (HR, 0.24, 95%CI, 0.11−0.53, *p* = .0007) (eFigure 2C).

Similar results were obtained if the prospective and retrospective older‐generation ASM cohorts were combined (*n* = 105) (eFigure 2D–F). The cumulative HRs of newer‐generation ASM were 0.54 (95%CI, 0.38−0.77, *p* = .0010) for ASM effectiveness, 0.43 (95%CI, 0.30−0.62, *p* < .0001) for ASM retention, and 0.28 (95%CI, 0.13−0.59, *p* = .0008) for ASM tolerability.

## 4 DISCUSSION

The present study demonstrated advantages of newer‐generation ASM in the secondary prevention of PSE. The latest guidelines recommend ASM treatment for secondary PSE prophylaxis (Holtkamp et al., 2017), as more than 70% of patients with a first unprovoked late seizure after stroke experience a subsequent seizure over the 10‐year follow‐up period (Hesdorffer et al., 2009). When considering initial ASM selection, however, there is no current consensus regarding the effectiveness of newer‐generation ASM for seizure control in PSE, largely due to insufficient evidence (Gilad, 2012; Xu, 2019). In a recent meta‐analysis (Brigo et al., 2018), only two randomized control trials specifically targeting PSE were included, one comparing LTG to CBZ (Gilad et al., 2007) and the other LEV to controlled‐release CBZ (Consoli et al., 2012). These studies showed lower incidence of AE with LTG and LEV than CBZ; however, any differences in seizure freedom were not identified, likely due to small sample sizes. While a large nationwide retrospective study from Taiwan demonstrated that VPA and newer‐generation ASM had fewer recurrences of PSE than PHT (Huang et al., 2015), it possessed inherent issues in diagnostic accuracy emanating from the use of health insurance database as the primary data source. Although the PROPOSE study is not a randomized control trial, it is based on accurate diagnoses, with established examinations by board‐certified specialists and reliable prospective follow‐up information.

Regarding focal seizure in adults (mainly due to stroke), the ILAE report recommends CBZ, LEV, PHT, and ZNS as initial monotherapy based on level A evidence (Glauser et al., 2013). In recent clinical studies, older‐generation ASM, such as CBZ, VPA, and PHT, were still being used (Hsieh & Huang, 2011; Johnell & Fastbom, 2011; Larsson et al., 2019; Pugh et al., 2008). The standard and new antiepileptic drugs (SANAD) trial comparing newer‐generation ASM with CBZ in patients with focal epilepsy demonstrated LTG was better tolerated, though seizure control was not different than other drugs (Marson et al., 2007). In the subgroup analysis of Keppra versus Older Monotherapy in Epilepsy Trial (KOMET), comparing the effectiveness of LEV with extended‐release VPA and controlled‐release CBZ in elderly patients with newly diagnosed epilepsy, LEV had more favorable tolerability profiles (Pohlmann‐Eden et al., 2016). These studies did not demonstrate superiority of seizure control using newer‐generation ASM; however, a survey of expert opinion across the United States reported preference for LTG and LEV over PHT, GBP, PH, and CBZ in the elderly population (Shih et al., 2017).

The current PROPOSE study showed that use of newer‐generation ASM was associated with approximately 50% reduction of seizure recurrence, compared to older‐generation ASM. Similar results were obtained in verification analysis using our retrospective cohort data. Previous meta‐analyses have revealed no significant superiority of seizure control in newly diagnosed focal epilepsy (Lattanzi et al., 2019) and epilepsy in elderly patients (Lattanzi et al., 2019). A plausible reason for the higher seizure recurrence with older‐generation ASM may lie in the lower maintenance dosage used for older‐generation ASM. In previous RCTs, CBZ was maintained at approximately 600 mg/day and VPA at 1000 mg/day, while in the PROPOSE study, the mean maintenance dosage as a monotherapy was 213 mg/day for CBZ and 471 mg/day for VPA (eTable 2). However, the dosages were similar for newer‐generation ASM in previous and our studies. It is likely that managing physicians in the current study acknowledged the old age of the enrolled patients (median, 75.5 years), the low body weight (mean, 56 kg) and the reportedly higher carrier rate of HLA‐A*31:01 (15% in Japanese vs. 5% in Caucasians), an established risk for carbamazepine‐induced AE, Stevens‐Johnson syndrome, and toxic epidermal necrolysis (Mushiroda et al., 2018). In a clinical setting, older‐generation ASMs are more likely to be underdosed than newer‐generation ASMs to avoid serious adverse effects because of the narrow safety range between effectiveness and toxicity. Hence, in the current study, the superiority of newer‐generation ASMs was difficult to conclude in terms of seizure control compared to older‐generation ASMs. Nevertheless, the serum concentration of CBZ was within therapeutic range in 81.3% of the patients who received CBZ (eTable 2), suggesting management of older‐generation ASM was acceptable. Moreover, the sensitivity analysis, which excluded subjects with inadequate ASM concentrations from the older‐generation ASM group, still showed better seizure control with newer‐generation ASMs (eFigure 1). Regarding other potential reasons, PSE is associated with an inherently higher risk of seizure recurrence compared to other neurological diseases. In a population‐based study of unprovoked seizures due to static brain lesions, PSE correlated with the highest frequency of seizure recurrence over 10 years (71.5% in stroke, 46.6% in traumatic brain injury, and 63.5% in central nervous system infection) (Hesdorffer et al., 2009). The high recurrence rate of PSE might have led to the statistical differences found in the current study. In addition, elderly stroke survivors tend to have multiple comorbidities that require concomitant drug use. Polypharmacy may also interfere with the efficacy of older‐generation ASMs, which are known to have drug*–*drug interactions. Moreover, CBZ, a CYP‐inducing ASM, could interfere with the efficacy of statins, consequently increasing lipid levels (Mintzer et al., 2018). Several studies have also demonstrated the risk of subsequent stroke was increased after the onset of epilepsy (Chang et al., 2014; Cleary et al., 2004). This may lie in the use of older‐generation ASM, which may affect the lipid profiles or drug metabolism of antithrombotic drugs. Older‐generation ASM may interfere with stroke prophylaxis and affect cerebrovascular risks (Zelano, 2016). Such effects may be important in the selection of ASM regimen in clinical practice in stroke survivors.

Our study has several limitations. First, as this was a prospective observational study, ASM regimen could not be controlled, meaning the older‐generation ASM were used less frequently and in smaller dosage than newer‐generation ASM. Second, the PROPOSE study was not a like‐for‐like comparison of each ASM, which may have resulted in insufficient statistical power. However, RCTs may potentially limit generalizability, which cannot be necessarily regarded as general principles valid for all subjects, some of whom may have had difficulties answering more complex questions. In the field of epilepsy, for instance, the various combinations of ASMs are used in a wide variety of clinical, social, and economic conditions. It is impractical to conduct separate RCTs to study the effect of each specific ASM regimen. Observational studies based on routinely collected data have generally a greater external validity than RCTs (Carlson & Morrison, 2009). Third, the diagnosis of PSE may be difficult due to the diverse semiology of PSE (Bentes et al., 2017). In order to increase reliability, we performed brain MRI, SPECT, and EEGs in the acute stage under careful consideration by certified epileptologists or neurologists.

## 5 CONCLUSIONS

The current study is a real‐world prospective cohort study to evaluate seizure control, retention, and tolerability of older or newer‐generation ASM treatment in PSE. These findings suggest potential for newer‐generation ASM as the primary choice in the secondary prophylaxis of PSE. However, further studies are needed to confirm ASM drug interactions and stroke risks and to find the optimal regimen for ASM in PSE.

## CONFLICT OF INTEREST

Akio Ikeda belongs to The Department of Epilepsy, Movement Disorders, and Physiology, an Industry‐Academia Collaboration Course, supported by a grant from Eisai Corporation, Nihon Kohden Corporation, Otsuka Pharmaceutical Co., and UCB Japan Co.

All other authors report no conflict of interest specifically related to this manuscript.

## AUTHOR CONTRIBUTIONS

*Study concept and design* was provided by TT, KF, KK, AS, KN, RM, AI, and MI. *Acquisition, analysis, or interpretation of data* was performed by TT, KF, and MI. *Drafting the manuscript and figures* was performed by TT and MI. All authors participated in critical revision of the article for intellectual content. KN and DO were the statisticians. AI and MI provided study supervision.

### PEER REVIEW

The peer review history for this article is available at https://publons.com/publon/10.1002/brb3.2330

## Supporting information

Supporting information

## Data Availability

Analyses for PROPOSE study are ongoing; however, once completed, the data generated from this work will be available upon reasonable request.
