# Supplementary material for: Antiseizure medications for post‐stroke epilepsy: A real‐world prospective cohort study
Source: Brain Behav. 2021 Aug 22;11(9):e2330. doi: 10.1002/brb3.2330 (PMC8442594; doi:10.1002/brb3.2330)
Supplement: Supplementary file 1 — Supporting information [file BRB3-11-e2330-s001.pdf]

Antiseizure medications for post-stroke epilepsy: a real-world prospective cohort study

Tomotaka Tanaka, MD[1], Kazuki Fukuma, MD[1], Soichiro Abe, MD[1], Soichiro Matsubara, MD, PhD[2], Rie Motoyama, MD[3], Masahiro Mizobuchi, MD, PhD[4, 14], Hajime Yoshimura, MD, PhD[5], Takayuki Matsuki, MD[6], Yasuhiro Manabe, MD, PhD[7], Junichiro Suzuki, MD, PhD[8], Shuhei Ikeda, MD[1], Naruhiko Kamogawa, MD[1], Hiroyuki Ishiyama, MD[1], Katsuya Kobayashi, MD, PhD[9], Akihiro Shimotake, MD, PhD[9], Kunihiro Nishimura, MD, PhD[11], Daisuke Onozuka, PhD[11], Masatoshi Koga, MD, PhD[12], Kazunori Toyoda, MD, PhD[12], Shigeo Murayama, MD, PhD[3], Riki Matsumoto MD, PhD[13], Ryosuke Takahashi MD, PhD[9], Akio Ikeda MD, PhD[10], Masafumi Ihara MD, PhD[1]; for the PROPOSE Study Investigators

1. Department of Neurology, National Cerebral and Cardiovascular Center, Osaka, Japan
2. Department of Neurology, Graduate School of Medical Sciences, Kumamoto University, Kumamoto, Japan
3. Department of Neurology, Tokyo Metropolitan Geriatric Hospital and Institute of Gerontology, Tokyo, Japan
4. Department of Neurology, Nakamura Memorial Hospital, Sapporo, Japan
5. Department of Neurology, Kobe City Medical Center General Hospital, Kobe, Japan
6. Department of Neurology, St. Mary's Hospital, Fukuoka, Japan
7. Department of Neurology, National Hospital Organization Okayama Medical Center, Okayama, Japan
8. Department of Neurology, Toyota Memorial Hospital, Toyota, Japan
9. Department of Neurology, Kyoto University Graduate School of Medicine, Kyoto, Japan
10. Department of Epilepsy, Movement Disorders and Physiology, Kyoto University Graduate School of Medicine, Kyoto, Japan
11. Departments of Preventive Medicine and Epidemiology, National Cerebral and Cardiovascular Center, Osaka, Japan
12. Department of Cerebrovascular Medicine, National Cerebral and Cardiovascular Center, Osaka, Japan
13. Division of Neurology, Kobe University Graduate School of Medicine, Kobe, Japan
14. Clinic of Minami-ichijyo Neurology, Sappro, Japan

Corresponding author:
Masafumi Ihara, MD, PhD
Department of Neurology, National Cerebral and Cardiovascular Center, 6-1 Kishibeshimmachi, Suita, Osaka 564-8565, Japan
Tel.: +81-6-6170-1070; Fax: +81-6-6170-1782; E-mail: ihara@ncvc.go.jp

eTable 1

ASM regimens of each group

ASM	n
**Older-generation ASM (prospective cohort) n=36**	
CBZ	23
VPA	7
PHT	2
CBZ+VPA	1
CBZ+PHT	1
CBZ+CZP	1
VPA+PB	1
**Mixed generation ASM (prospective cohort) n=50**	
CBZ+LEV	17
CBZ+LTG	1
CBZ+ZNS	1
CBZ+PER	1
CBZ+PHT+GBP	1
CBZ+PHT+LEV	1
CBZ+LEV+TPM	1
VPA+LEV	9
VPA+ZNS	3
VPA+LEV+LCM	2
PHT+LEV	7
PHT+LCM	1
PHT+LEV+LCM	1
CZP+LEV	2
PB+LEV	1
ZNP+LEV+CLB	1
**Newer-generation ASM (prospective cohort) n=286**	
LEV	250
LCM	13
ZNS	2
LTG	5
GBP	1
LEV+LCM	5
LEV+PER	3
LEV+ZNS	3

LEV+GBP	1
LEV+TPM	1
LTG+ZNS	1
PER+ZNS	1
**Older-generation ASM (retrospective cohort) n=69**	
CBZ	25
VPA	21
PHT	18
VPA+PHT	3
VPA+CBZ	1
VPA+CZP	1

Abbreviations: ASM, antseizure medication; PER, perampanel; CBZ, carbamazepine; CZP, clonazepam; LEV, levetiracetam; LCM, lacosamide; LTG, lamotrigine; PB, phenobarbital; VPA, valproate; ZNS, zonisamide; CLB, clobazam; PHT, phenytoin; GBP, gabapentin; CZP, clonazepam; TPM, topiramate.

eTable 2

Mean daily dosage of ASM and proportion of adequate serum concentration of older-generation ASM at discharge*

ASM	Monotherapy ASM (mg/day)	Adequate serum ASM concentration (%)	Polytherapy ASM (mg/day)	Adequate serum ASM concentration (%)
**Older-generation ASM**				
CBZ (n=26)	213.0 ±92.0	81.3%	233.3 ±57.7	100%
VPA (n=10)	471.4 ±170.4	28.6%	800.0 ±0	100%
PHT (n=3)	200.0 ±0	0%	250.0 ±0	100%
PB (n=3)	-	-	60.0 ±0	-
CZP (n=1)	-	-	0.5 ±0	-
**Newer-generation ASM**				
LEV (n=263)	961.0 ±402.3	-	1384.6 ±704.3	-
LCM (n=18)	123.1 ±43.9	-	160.0 ±54.8	-
ZNS (n=7)	200.0 ±0	-	260.0 ±89.4	-
LTG (n=6)	95.0 ±67.1	-	150.0 ±0	-
PER (n=4)	-	-	4.0 ±1.6	-
GBP (n=2)	800.0 ±0	-	300.0 ±0	-
TPM (n=1)	-	-	200.0±0	-

Data presented as mean ±standard deviation.

*Information of adequate serum concentration ASM of 10 cases in older-generation ASM could not be obtained.

Abbreviations: ASM, antiseizure medication; PER, perampanel; CBZ, carbamazepine; CZP, clonazepam; LEV, levetiracetam; LCM, lacosamide; LTG, lamotrigine; PB, phenobarbital; VPA, valproate; ZNS, zonisamide; PHT, phenytoin; GBP, gabapentin; CZP, clonazepam; TPM, topiramate.

eTable 3

Drug-related adverse effects causing dosage adjustment or discontinuation

	Older-generation ASM n=36		Newer-generation ASM n=286	
	Dosage adjustment	Discontinuation	Dosage adjustment	Discontinuation
Skin eruptions	0	1	5	6
Somnolence	1	0	0	0
Dizziness	1	0	3	1
Behavioral changes	1	1	0	3
Abdominal symptoms	0	0	0	2
Liver enzymes increase	0	1	0	1
Others	0	1	0	0
total	3 (8%)	4 (11%)	8 (2.8%)	13 (4.5%)

Abbreviations: ASM, antiseizure medication.

eTable 4

Patient characteristics of older-generation ASM (retrospective cohort)

	**Older-generation ASM n=69**
Age, years, median (IQR)	72 (65-79)
Female (%)	20 (29)
Body weight, kg, median (IQR)	57 (50-65)
Current alcohol consumption (%)	7 (10)
Family history of epilepsy (%)	1 (1)
Previous PSE (%)	30 (43)
Previous ASM (%)	23 (33)
Comorbidities	
Hypertension (%)	54 (78)
Dyslipidemia (%)	30 (43)
Diabetes (%)	16 (23)
Chronic kidney diseases (%)	31 (45)
Liver cirrhosis (%)	1 (1)
Atrial fibrillation (%)	26 (38)
Dementia (%)	25 (36)
Craniotomy (%)	10 (14)
Etiology of stroke subtype (%)	
Infarction (%)	49 (71)
Cardiac embolism (%)	22 (32)
Hemorrhage (%)	22 (32)
TIA (%)	1 (1)
SAH (%)	3 (4)
Previous early seizure* (%)	2 (4)
Stroke lesion	
Frontal lobe (%)	13 (19)
Parietal lobe (%)	27 (39)
Temporal lobe (%)	25 (36)
Occipital lobe (%)	14 (20)
Cortical lesion (%)	51 (74)
Stroke size	
<15 mm (%)	16 (23)
15-30 mm (%)	14 (20)
>30 mm (%)	39 (57)
Clinical course during hospitalization	

Classification of seizures	
Focal aware seizure (%)	6 (9)
Focal impaired awareness seizure (%)	21 (30)
Focal-to-bilateral tonic-clonic seizure (%)	42 (61)
Others (%)	0 (0)
Detail of seizure	
Focal or generalized motor seizure (%)	49 (71)
Weakness (%)	9 (13)
Aphasia (%)	15 (22)
Amnesia (%)	1 (1)
EEG findings (n=62)	
Focal slow wave (%)	36 (58)
Spike/sharp wave (%)	16 (26)
Rhythmic slow wave (%)	9 (15)
Periodic discharge (%)	4 (6)
Hyperperfusion on SPECT (%) (n=48)	33 (69)
Seizure-related hyperintensity on DWI (%) (n=64)	17 (27)
Hospitalization period, days, median (IQR)	11 (8-16)
mRS at discharge, median (IQR)	2 (1-4)
ASM combination therapy (%)	5 (7)

Abbreviations: ASM, antiseizure medication; IQR, interquartile range; PSE, post-stroke epilepsy; TIA, transient ischemic attack; SAH, subarachnoid hemorrhage; NIHSS, National Institutes of Health Stroke Scale; EEG, electroencephalogram; SPECT, single photon emission computed tomography; DWI, diffusion weighted image; mRS, modified Rankin Scale

Data are presented as median (interquartile range) or absolute (percentage) values.

*The history of early seizure was not available in 22 cases due to earlier admission in a different hospital.

eFigure 1

Kaplan-Meier curves of prospective newer-generation ASM (n=286) vs. prospective older-generation ASM (n=18) for seizure recurrence after eliminating subjects with inadequate or unknown serum concentration for older-generation ASM (n=18)

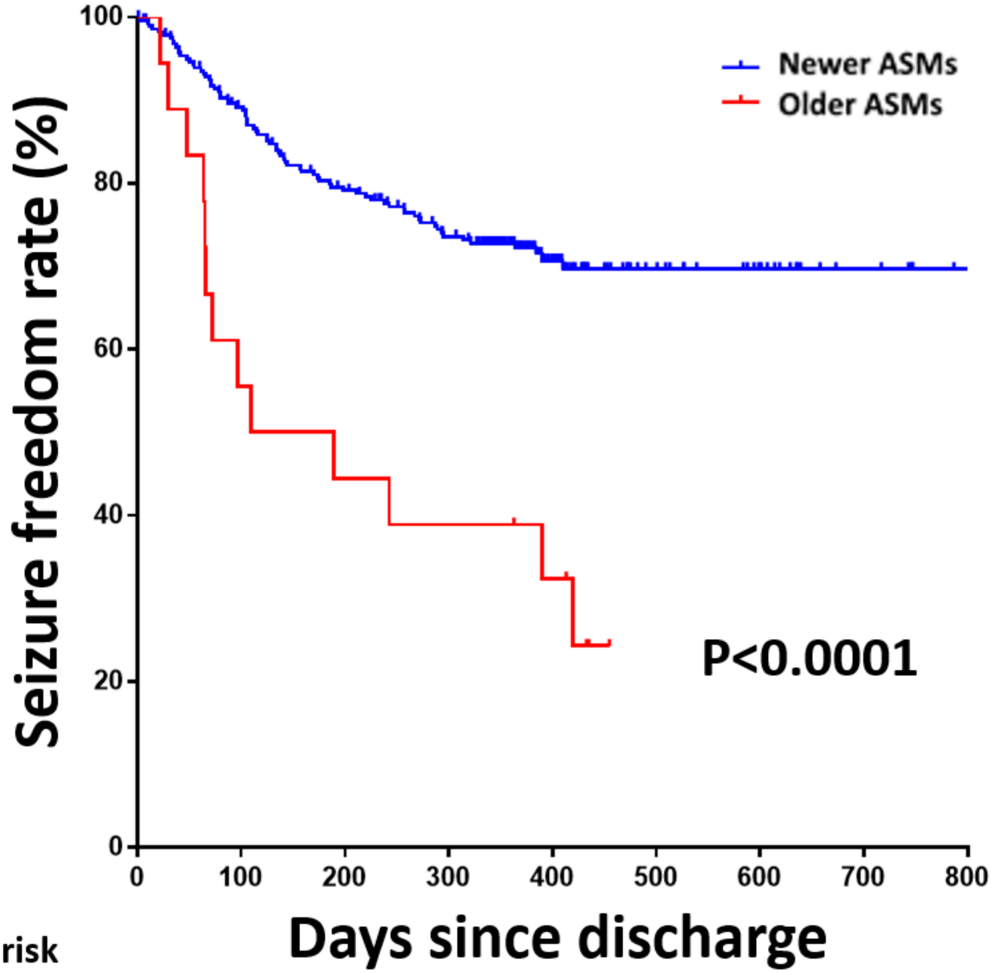

Number at risk

	0	100	200	300	400	500	600	700	800
Newer ASMs	286	241	209	180	76	33	21	11	0
Older ASMs	18	11	9	8	6	0	0	0	0

eFigure2

Verification results of Kaplan-Meier curves for seizure recurrence, retention, and tolerability of ASM

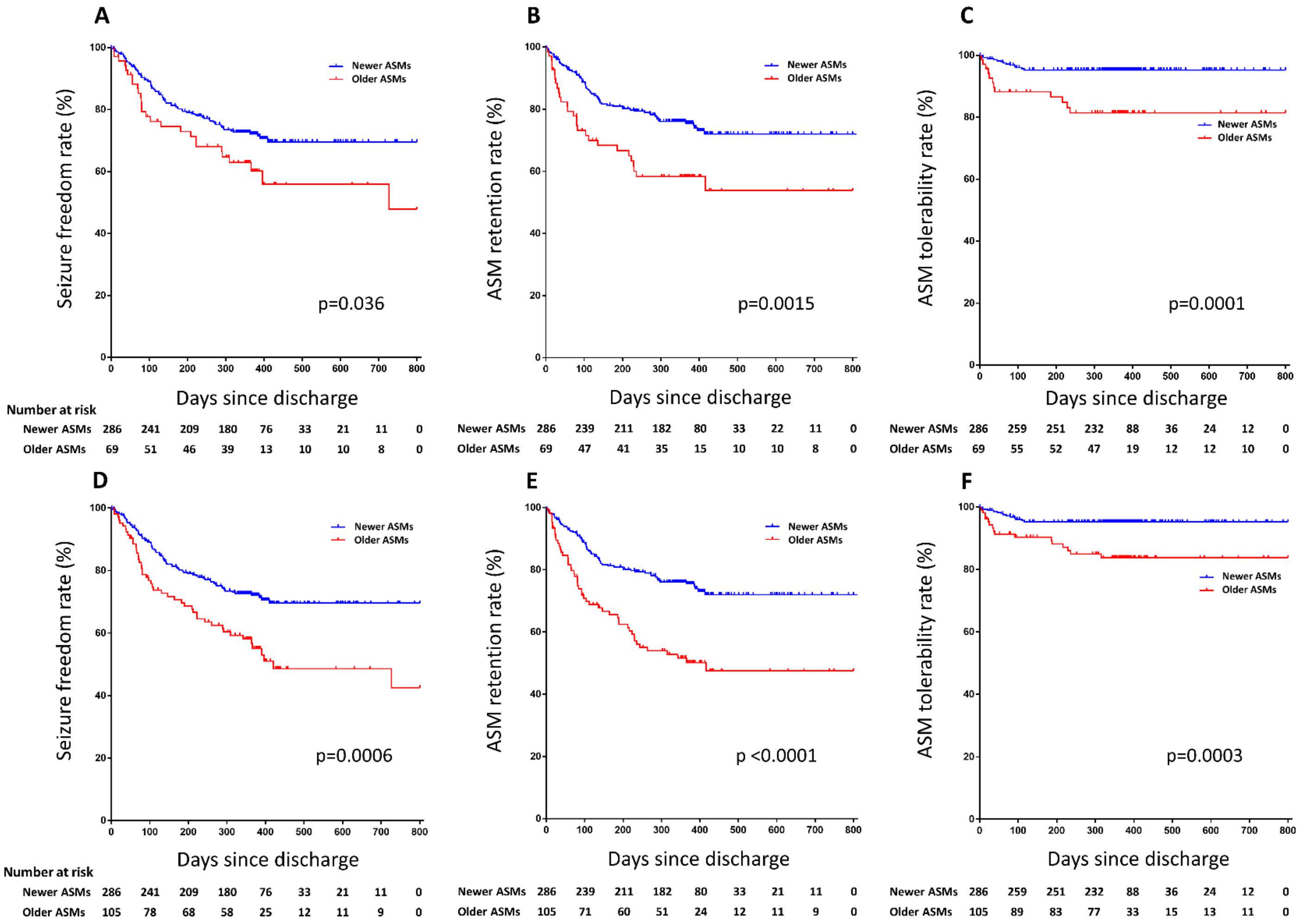

(A-C) Kaplan-Meier curves of prospective newer-generation ASM (n=286) vs. retrospective older-generation ASM (n=69) for (A) time to seizure recurrence, (B) ASM discontinuation and dosage adjustment (retention), and (C) time to discontinuation of initial ASM regimen (tolerability).

(D-F) Kaplan-Meier curves of prospective newer-generation ASM (n=286) vs. prospective & retrospective older-generation ASM (n=105) for (D) time to seizure recurrence, (E) ASM discontinuation and dosage adjustment (retention), and (F) time to discontinuation of initial ASM regimen (tolerability).

Censored values are indicated by dots.
Mixed generation ASM group (n=50) was excluded in the analyses.
